# Selection for depression-specific dementia cases with replication in two cohorts

**DOI:** 10.1371/journal.pone.0216413

**Published:** 2019-05-31

**Authors:** Donald R. Royall, Raymond F. Palmer, for the Alzheimer’s Disease Neuroimaging Initiative (ADNI) and the Texas Alzheimer’s Research and Care Consortium (TARCC)

**Affiliations:** 1 Department of Psychiatry, the University of Texas Health Science Center, San Antonio, Texas, United States of America; 2 Department of Medicine, the University of Texas Health Science Center, San Antonio, Texas, United States of America; 3 Department of Family and Community Medicine, the University of Texas Health Science Center, San Antonio, Texas, United States of America; 4 South Texas Veterans’ Health System Audie L. Murphy Division Geriatric Research Education and Clinical Care Center, San Antonio, Texas, United States of America; Nathan S Kline Institute, UNITED STATES

## Abstract

The latent variable “δ” (for “dementia”) provides an etiologically “agnostic” omnibus dementia severity metric capable of recognizing the dementing potential of any condition. Depressive symptoms are independent predictors of δ and are thereby implicated as “dementing”. Serum resistin levels partially mediate the association between depressive symptoms and δ. We use a novel “off-diagonal” CHI SQ algorithm to demonstrate our ability to select individuals demented solely by depression’s effect in both the Texas Alzheimer’s Research and Care Consortium (TARCC) (N ≌ 3,500), and the Alzheimer’s Disease Neuroimaging Initiative (ADNI (N ≌ 1,750), and demonstrate the higher resistin levels of such cases in TARCC. This approach can be adapted to any δ-related dementia risk factor or biomarker and used identify individuals who might revert back to non-demented states after its successful treatment.

## Introduction

Using theory-driven confirmatory bifactor analyses (CFA) in a Structural Equation Model (SEM) framework, we have discovered a transdiagnostic omnibus dementia severity measure (i.e., “δ” for “dementia”) [1]. δ represents “the cognitive correlates of functional status”. By definition, it embodies cognition’s association with instrumental activities of daily living (IADL) and it is empirically strongly related to dementia severity [as measured by the Clinical Dementia Rating scale “Sum of Boxes”(CDR-SB)] [2], both cross-sectionally and longitudinally [1, 3–4].

δ’s strong association with clinical dementia has been independently replicated in the National Alzheimer’s Coordinating Center (NACC)’s Uniform Dataset (UDS) (N = 26,606) [3] and in well characterized European [5] and Austral-Asian [6–7] samples. However, these studies also reveal δ to be “agnostic” to dementia’s etiology. δ has a high AUC for the diagnosis of all cause dementia in the NACC (i.e., 0.96) [1], but it cannot distinguish between any two dementia etiologies [8]. Thus, δ does not convey etiologically-salient information. Etiologically-specific information is conveyed instead by domain-specific cognitive factors, residual (and therefore unrelated) to δ. δ cannot distinguish any two dementias because *it* is dementia’s essential cognitive impairment.

δ can be “reified” as a factor composite and assigned to individuals as a “d-score”. Because d-scores are continuously distributed, δ effectively converts dementia from a *category* to a *dimension*. It can thereby rank order, individuals, even normal controls (NC), with regard to their dementia “severity” and /or equate them for severity across diagnoses. Even slight differences in the d-scores of persons without dementia increases their prospective risk of conversion to clinical dementia [9].

δ is derived from Spearman’s general intelligence factor “*g*” [10]. In consequence, it must be distinguished from domain-specific cognitive performance [i.e., “memory” (MEM) or “executive function” (EF)] [11]. This both undermines the latter’s claims of contribution to IADL impairment and implicates a disruption of *g* as dementia’s essential feature.

*g* manifests in every cognitive performance measure. Since δ is derived from *g*, it too appears to be estimable from any cognitive battery that contains a measure of IADL. δ can be constructed from a comprehensive battery of formal measures [1, 3, 5–6, 8], from small batteries of formal measures [12–13], from brief batteries of “bedside” measures [14], and even from the items of a single measure [15]. Thus, we must further distinguish between δ i.e., “the cognitive correlates of functional status”, and “d” i.e., δ’s “reification” as a composite “d-score” in a specific cognitive battery or analysis. So many batteries are available that we refer to each embodiment as a δ “homolog”. In genetics, a homolog is a gene descended from an ancestral gene in the same species and preserving the original’s function. All δ homologs (fourteen published to date) share δ’s bifactor construction, target a measure of IADL, exhibit strong associations with dementia severity (e.g., as measured by CDR-SB) and achieve high areas under the receiver operating characteristic curve (AUC /ROC) for the discrimination of various dementias from NC.

Given δ’s unique association with dementia, dementia’s risk factors must be associated with δ and δ’s biomarkers are likely to mediate dementing processes. Age, depression, and the apolipoprotein E (APOE) e4 allele are independently associated with δ [16]. We have reported the serum protein biomarkers that mediate each of their unique associations with δ [17–19]. Independently of those risk factors, δ is associated with additional pro- and anti-inflammatory serum protein biomarkers, [16, 20–22]. It is unclear how these might relate to traditional Alzheimer’s Disease (AD)-specific biomarkers, but it is more clear that unless the latter can also be associated with δ, their effects on cognitive performance will not be dementing.

It seems increasingly likely then that dementia severity, as estimated by δ homologs, represents the summed effects of multiple independent δ-related processes. There may be interindividual variability with regard to which specific processes are responsible for the observed d-score in an individual patient. Each process may have its own unique set of biomarkers and yet explain only a portion of δ’s variance. It becomes desirable then to intervene on the processes that are most amenable to treatment and /or contribute most to the individual’s d-score. In individual patients, the treatable subset may be entirely responsible for the observed d-score, or only a minor component. Thus, the impact of successful intervention may depend on the targeted process’ contribution to the observed d-score. Ideally, we would want to intervene on patients who might revert back across the d-score threshold for “clinical” dementia into δ’s non-demented range. The goal is to select patients who are not merely afflicted by the targeted dementing process, but who have been pushed over the d-score threshold for clinical dementia solely by that process. Moreover, such cases will have to be identified as individuals so that they can be targeted precisely for treatment.

### Approach to case selection

**Fig 1:**
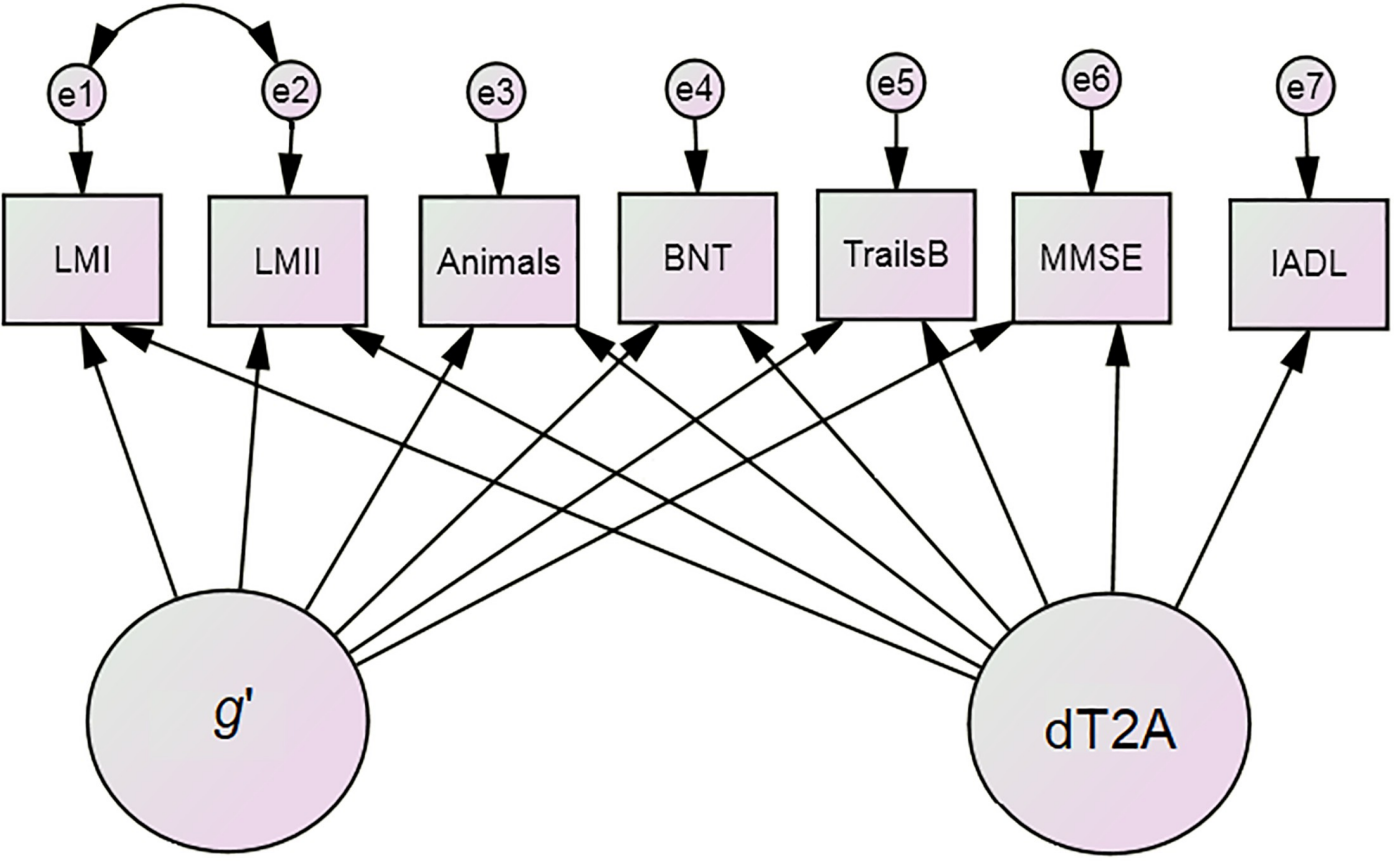
dT2A.

We propose the following algorithm to detect individuals most likely to respond to an intervention against any pre-specified dementing process (e.g., depressive symptoms in the present analysis). By definition, all dementing processes must impact δ. While any δ homolog might be employed, we chose the recently validated “dT2A” (TARCC to ADNI) homolog. “ADNI” refers to the Alzheimer’s Disease Neuroimaging Initiative (ADNI). dT2A was specifically engineered to replicate across the TARCC and ADNI studies [23]. We chose this homolog because we intend to validate the cases selected by this approach on the basis of certain blood-based protein biomarkers (e.g., resistin) which we have previously shown to mediate the unique association between δ and depressive symptoms [19]. The dT2A homolog (Fig 1) has been reported to have excellent fit in both datasets [TARCC: CHI SQ = 73.6 (20), p < 0.001; CFI = 0.996; RMSEA = 0.028; ADNI: CHI SQ = 12.464 (7), p < 0.001; CFI = 0.999; RMSEA = 0.019], to correlate strongly with CDR-SB in both datasets [TARCC: r = 0.99, p<0.001; ADNI: r = 0.96, p<0.001], and to achieve a high AUC for AD’s discrimination from NC [TARCC: AUC = 0.981 (0.976–0.985); ADNI: AUC = 1.0 (0.995–1.00)] [23].

For the present analysis, we chose depressive symptoms as the δ-related risk factor of interest because of the widespread availability of effective antidepressant treatments. We propose to identify individuals in TARCC who have converted to clinical dementia, nominally “AD”, solely by the effect of depressive symptoms on their d-scores, and to validate the selected cases on the basis of biomarkers known to mediate that specific association.

First, we must reconstruct dT2A in both cohorts. No covariate adjustments are applied to the raw data. Next, we adjust dT2A for depressive symptoms. To that end, we constructed a dichotomous dummy variable (GDS-di) coding scores ≥11/30 on the GDS, i.e., at the GDS’ recommended threshold for “clinical depression” [24]. This divides both samples into cases with higher and lower burdens of depressive symptoms. By adjusting only for depressive symptoms, the variance related to all other δ-related dementia risks remains in the composites.

Next, we identify the thresholds for dementia conversion by both the adjusted and unadjusted composites, in both datasets, by ROC analysis. The thresholds are all set at Specificity = 0.85. Next, we dichotomize each cohort into “demented” and “non-demented” cases, by both the adjusted and unadjusted composites.

Since the adjusted and unadjusted composites differ only with regard to the variance related to depressive symptom burden, they are expected to be highly correlated, *but not identical*. To confirm this, we regress the adjusted and unadjusted dT2A composites. Because they are not identical, there is expected to be some discrepancy in the classifications derived from these composites. By comparing the resulting adjusted and unadjusted classifications in a CHI-SQ table, “off-diagonal” cases can be recognized as individuals. These represent individuals whose classification changes when depression’s unique effect on δ is considered. The group which reverts back to a non-demented d-score after the GDS’ impact has been adjusted can be considered to have been demented solely on the basis of their depressive symptom burden. To confirm this conclusion, we contrast serum (TARCC) and plasma (ADNI) resistin levels across the off-diagonal groups. This approach also allows us to estimate the fraction of so-called “AD” cases who have been demented (i.e., pushed across dT2A’s threshold for clinical conversion) by a GDS score ≥11 in both the TARCC and ADNI cohorts.

## Materials and methods

The samples and methods employed in dT2A’s construction and in the present analysis are identical to Royall & Palmer, 2018 [23] and reiterated here for the convenience of the reader.

### Subjects

The present study is a secondary analysis of data collected by TARCC and ADNI. Informed consent was obtained from all participants (or their legally authorized proxies) before data collection, and both studies are approved by their respective Institutional Review Boards (IRB).

#### TARCC

Subjects included N = 3502 Texas Alzheimer’s Research and Care Consortium (TARCC) participants [25]. TARCC is a longitudinally followed convenience sample of elderly persons with Alzheimer’s disease (AD) (n = 1275), “Mild Cognitive Impairment “(MCI) (n = 732), or normal cognition (NC) (n = 1445) (and 58 “others”) recruited from five Texas medical schools. Each participant underwent a standardized annual examination that included a medical evaluation, neuropsychological testing, and clinical interview. Categorical clinical diagnoses of “AD”, “MCI” and “NC” were established through consensus. The diagnosis of AD was based on National Institute for Neurological Communicative Disorders and Stroke-Alzheimer’s Disease and Related Disorders Association (NINCDS-ADRDA) criteria [26]. The diagnosis of MCI was based on site-specific consensus-based clinical diagnoses derived from all available information but without reliance on specific neurocognitive tests and /or cut-scores. “All available information” included the results of TARCC’s entire neuropsychological battery, clinical evaluations, informant interviews, and any available outside medical records. We could not easily use cut-scores because normative Mexican-American (MA) scores are not available for many measures.

#### ADNI

ADNI is a well-characterized longitudinal convenience sample developed to validate the magnetic resonance, positron emission tomography (PET), cerebrospinal fluid (CSF), and genetic biomarkers of AD [27]. The initial 5-year study, ADNI-1, enrolled cognitively normal, mild cognitive impairment (MCI) and AD subjects, and subsequent studies (ADNI-GO and ADNI-2) added early- and late-MCI cohorts. ADNI has provided a framework for similar initiatives worldwide, including TARCC. In its combined sample (N = 1738), N = 342 were diagnosed with AD, N = 978 with MCI and N = 417 as NC. For this analysis, all MCI subtypes were combined, including ADNI-GO participants with “Subjective cognitive impairment (SCI)”.

### Clinical variables

#### dT2A, a δ homolog for ADNI

dT2A is indicated by observed cognitive measures that are common to both TARCC and ADNI, including the Boston Naming Test (BNT) [28], Category Fluency (Animals) [29], Logical Memory I (LMI) and II (LMII) [30], the Mini-Mental State Examination (MMSE) [31], and Trail-Making Part B (TrailsB) [32]. All are available in TARCC in Spanish translation.

Boston Naming Test (BNT) [28]: The BNT is a confrontation naming test that requires the subject to verbally name line drawings of objects associated with words of increasingly lower frequency in the target language. TARCC uses 30 item BNT. ADNI uses 60 item BNT.

Categorical Fluency (Animals) [29]: This test of verbal fluency asks subjects to verbally generate as many animal names as they are able in one minute.

Logical Memory II (LMII) [30]: Immediately (LMI), and following a thirty minute delay (LMII), the subject recalls two paragraphs read aloud.

Mini-Mental Status Examination (MMSE) [31]: The MMSE is a well-known and widely used test for screening cognitive impairment.

Trail Making Part B (Trails B) [32]: Trails B is a timed test of attention, speed, and mental flexibility that requires the subject to alternately connect between numbers and letters. TARCC reports Trails B as scaled scores.

#### dT2A’s target indicators

In TARCC, we used informant-rated Instrumental activities of daily living (IADLs) [33] as dT2A‘s target indicator. Unfortunately, IADL is not available in ADNI, and so the Functional Assessment Questionnaire (FAQ) [34] was used instead. The FAQ has been successfully incorporated into δ homologs by other investigators [10–11].

Instrumental Activities of Daily Living: IADL was assessed using Lawton’s method [33]. This involves a structured clinical interview that provides informant-reported information on seven IADLs. Each item is scored on a four point Likert scale with 0 signifying “no impairment”.

The Functional Activities Questionnaire (FAQ) [34]: The FAQ is an informant-rated measure of a participant’s ability to be perform IADLs. The FAQ is commonly used in dementia evaluations [35–36].

#### Observed clinical measures

Observed clinical measures are often used as covariates or to provide external validation. The following measures are available in both TARCC and ADNI.

Self (informant)-reported age, and gender are self-explanatory. Education was measured in years. Ethnicity is coded dichotomously according to self-reported Hispanic affiliation. TARCC has a substantial number of MA participants. MA ethnicity has pronounced effects on serum protein biomarkers in TARCC [22, 37]. There are no racial distinctions in TARCC, and no reported racial effects on plasma protein biomarkers in ADNI.

The Clinical Dementia Rating Scale “Sum of Boxes” (CDR-SB) [2]. The CDR is used to evaluate dementia severity. The rating assesses the patient’s cognitive ability to function in six domains–memory, orientation, judgment and problem solving, community affairs, home and hobbies and personal care. Information is collected during an interview with the patient and their caregiver (15 minutes).

Geriatric Depression Scale (GDS): Depressive symptoms were assessed in both studies by the Geriatric Depression Scale (GDS) [24, 38]. GDS scores range from zero-30. Higher scores are worse. The GDS is valid in persons with dementia [39].

### Statistical analyses

These analyses were conducted in TARCC’s most recent dataset (N = 3502) and in a combined sample of ADNI-1, ADNI-2, and ADNI-GO data (N = 1737). The analysis was performed using Analysis of Moment Structures (AMOS) software [40]. The maximum likelihood estimator was chosen for these models. Covariances between the residuals were allowed to be estimated if they were significant and improved model fit.

The observed variables were fit to a linear confirmatory bifactor model. Measurement errors are assumed uncorrelated and the latent variables means and variances were fixed to 0 and 1 respectively allowing all loadings to be freely estimated.

#### Missing data

We used Full Information Maximum Likelihood (FIML) methods to address missing data. FIML uses the entire observed data matrix to estimate parameters with missing data. In contrast to listwise or pairwise deletion, FIML yields unbiased parameter estimates and preserves the overall power of the analysis [41–42].

#### Fit indices

The validity of structural models was assessed using two common test statistics. A non-significant chi-square signifies that the data are consistent with the model [43]. However, with large samples chi-square will often be significant, even for models which fit the data well. Therefore, the ratio of the chi-square to the degrees of freedom in the model is also of interest. A CMIN/DF ratio < 5.0 suggests an adequate fit to the data [44]. The comparative fit index (CFI), with values ranging from between 0 and 1, compares the specified model with a model of no change [45]. CFI values below 0.95 suggest model misspecification. Values of 0.95 or greater indicate adequate to excellent fit. A root mean square error of approximation (RMSEA) of 0.05 or less indicates a close fit to the data, with models below 0.05 considered “good” fit, and up to 0.08 as “acceptable” [46]. All three fit statistics should be simultaneously considered to assess the adequacy of the models to the data.

## Results

Descriptive statistics are presented by group in Table 1. Cohen’s d and t tests of significance are reported in Table 1, where estimable, for TARCC vs. ADNI. These samples differed significantly on all measures. ADNI appears to have a relatively high fraction of MCI cases, which were recruited explicitly into ADNI-2 and ADNI-GO. TARCC has a much higher prevalence of MA participants.

**Table 1:** Descriptive statistics by sample (raw scores except where indicated).

	TARCC Total N = 3502	ADNI N = 1738
	**Mean (SD)**	**Mean (SD)**
**AD cases**	1275 (37.0%)	342 (19.7%)
**MCI cases**	723 (21.0%)	978 (56.3%)
**NC**	1445 (41.9%)	417 (24.8%)
**Gender (%♀)**	61.6	55.1
**Ethnicity (%MA)**	35.7	3.4
	**Mean (SD)**	**Mean (SD /d1)**
**Age**	70.8 (9.6)	73.8 (7.19 / 0.35^‡^)
**Education**	13.3 (4.3)	15.91 (2.86 / 0.71^‡^)
**MMSE**	25.6 (4.7)	27.17(2.67 / 0.42^‡^)
**Animals**	14.9 (5.5)	17.15 (5.93 / 0.39^‡^)
BNT*	7.9 (4.3)	25.97 (4.51 / **)
**CDR-SB**	2.4 (3.3)	1.64 (1.79 / 0.28^‡^)
**GDS30**	5.6 (5.2)	1.42 (1.40 / 1.09^‡^)
**LMI**	7.9 (4.2)	9.28 (4.83 / 0.30^‡^)
**LMII**	8.2 (4.6)	7.07 (5.33 / 0.22^‡^)
**Trails B (sec)**	144.24 (84.05)	122.23 (75.78 / 0.27^‡^)

d1 = Cohen’s d vs. TARCC’s entire sample.

*Scaled scores.

**TARCC uses 30 item BNT, ADNI uses 60 item BNT.

^‡^ p <0.001

ADNI = Alzheimer’s Disease Neuroimaging Initiative; Animals = Animal Naming; BNT = Boston Naming Test; CDR-SB = Clinical Dementia Rating scale “Sum of Boxes”; GDS = 30 item Geriatric Depression Scale; LMI = Wechsler Logical Memory immediate recall; LMII = Wechsler Logical Memory delayed recall; MA = Mexican-American; MMSE = Mini-mental State Exam; SD = standard deviation; TARCC = Texas Alzheimer’s Research and Care Consortium; Trails B = Trail Making Test Part B.

Descriptive statistics by diagnosis are provided for TARCC and ADNI (respectively) in Tables A and B in S1 File.

dT2A’s unadjusted model had excellent fit in both cohorts as previously reported [17]. The GDS adjusted models also fit well [i.e., TARCC: CHI SQ = 50.9 (7), p < 0.001; CFI = 0.998; RMSEA = 0.042; ADNI: CHI SQ = 71.7 (7), p < 0.001; CFI = 0.994; RMSEA = 0.064]. The GDS-adjusted composites achieved high Areas Under the Receiver Operating Characteristic Curve (AUC /ROC) for AD’s discrimination from NC [i.e., TARCC: AUC = 0.964 (0.976–0.985); ADNI: AUC = 0.988 (0.983–0.993)]. At a threshold of -0.0441, the adjusted composite had a sensitivity of 0.974 and a specificity of 0.845 for AD’s diagnosis in TARCC. In ADNI, a threshold of -0.068 on the adjusted composite had a sensitivity of 0.959 and a specificity of 0.845 for AD’s diagnosis.

**Fig 2:**
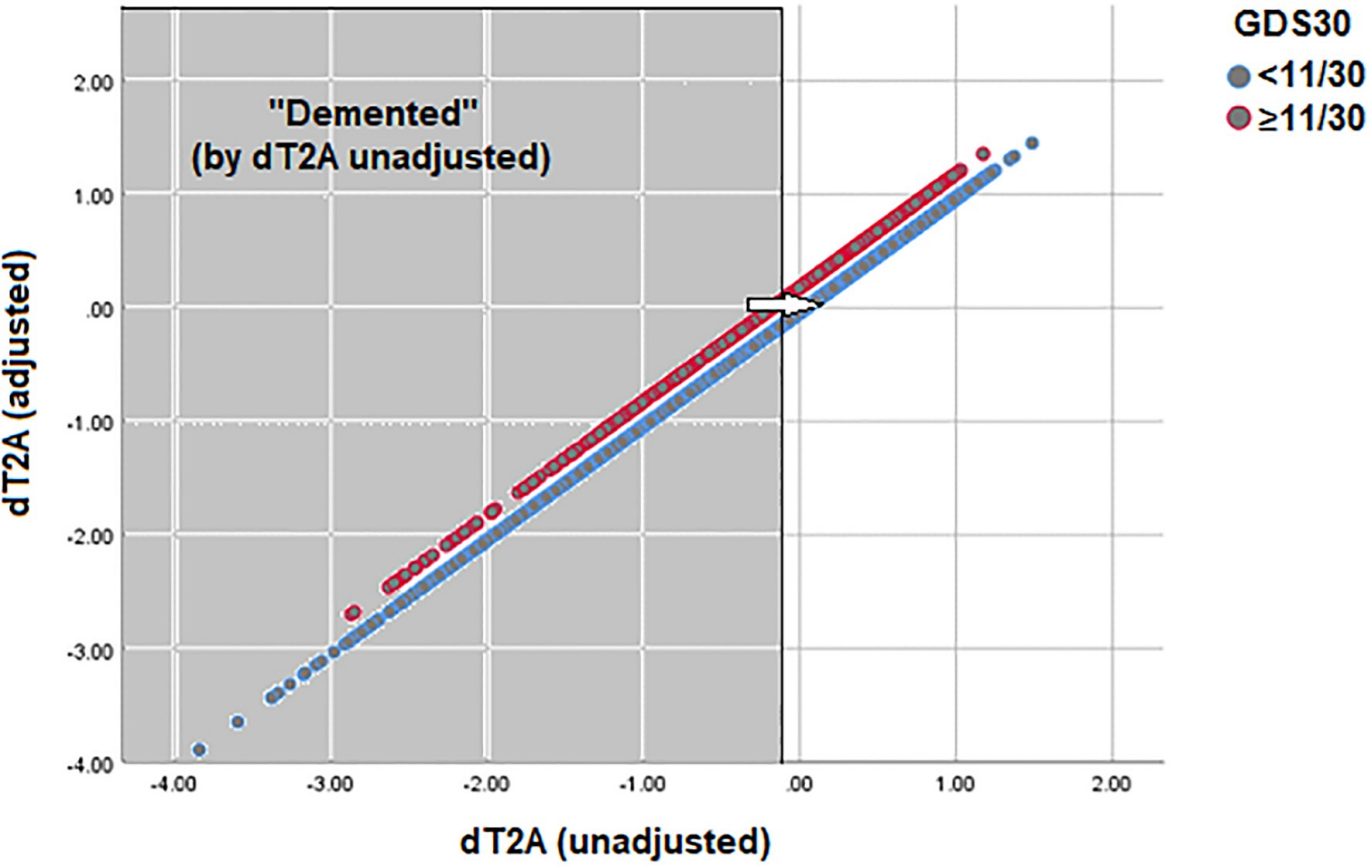
Scatterplot of GDS-adjusted vs. unadjusted dT2A scores (Grouped by GDS ≥11/30)*. * Note right shifted distribution (away from dementia) in non-depressed cases. GDS = Geriatric Depression Scale; GDS Scores have been dichotomously split at GDS ≥11/30.

The GDS-adjusted and unadjusted composites were strongly correlated in both datasets (i.e., TARCC: r = 0.987, p <0.001; ADNI: r = 0.998, p <0.001). Both regressions exhibit GDS effects on dT2A. Fig 2 presents the TARCC data. A high GDS score effectively left-shifts δ’s association with its GDS adjusted composite along their entire range. This suggests that depressive symptoms even increase the “dementia severity” of persons without dementia by placing them closer to δ’s critical threshold for dementia conversion. Similar results are seen in ADNI (data not shown).

**Fig 3:**
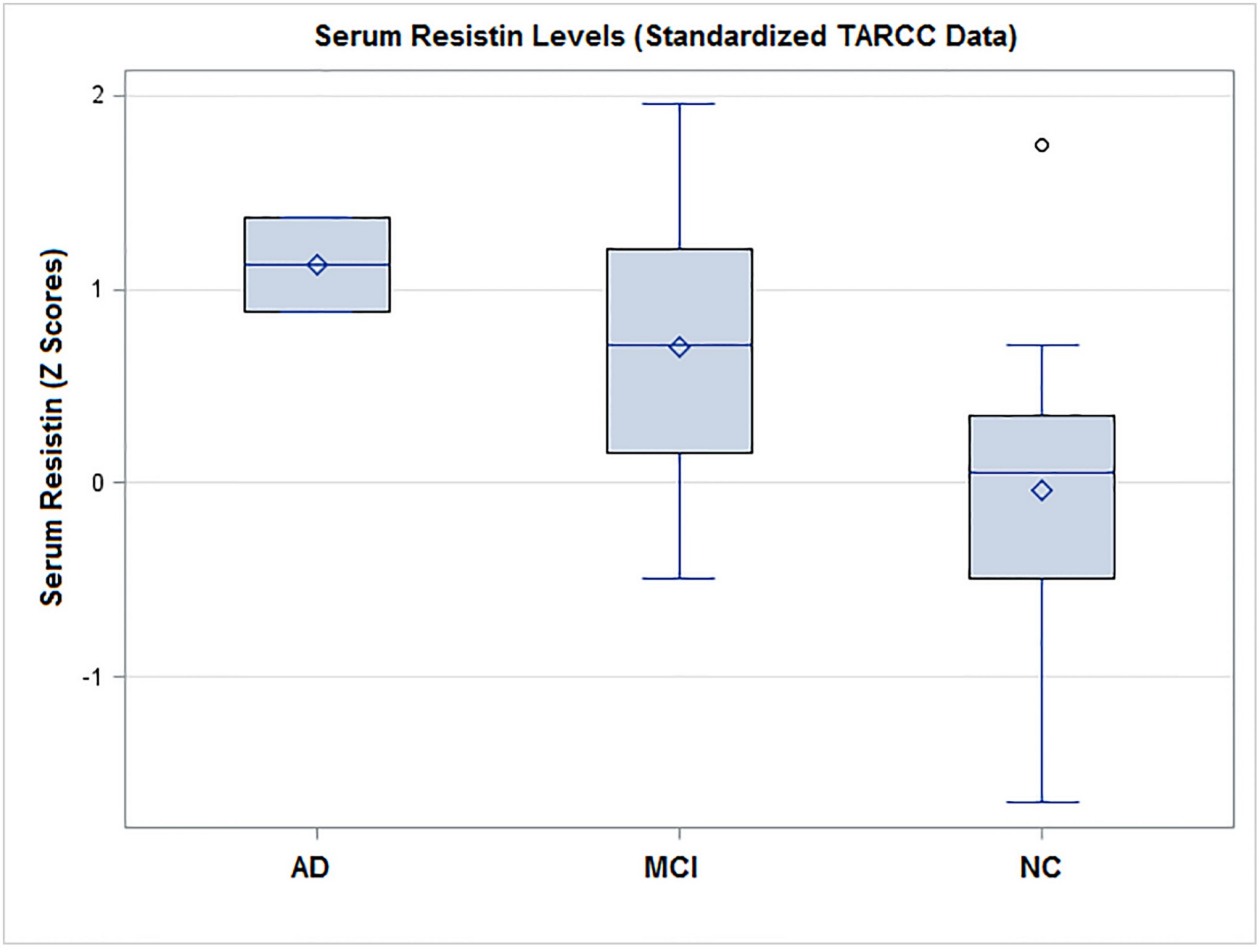
Serum resistin levels are elevated in AD cases (TARCC). *AD = Alzheimer’s Disease; MCI = Mild Cognitive Impairment; NC = Normal Controls; TARCC = Texas Alzheimer’s Research and Care Consortium.

**Fig 4:**
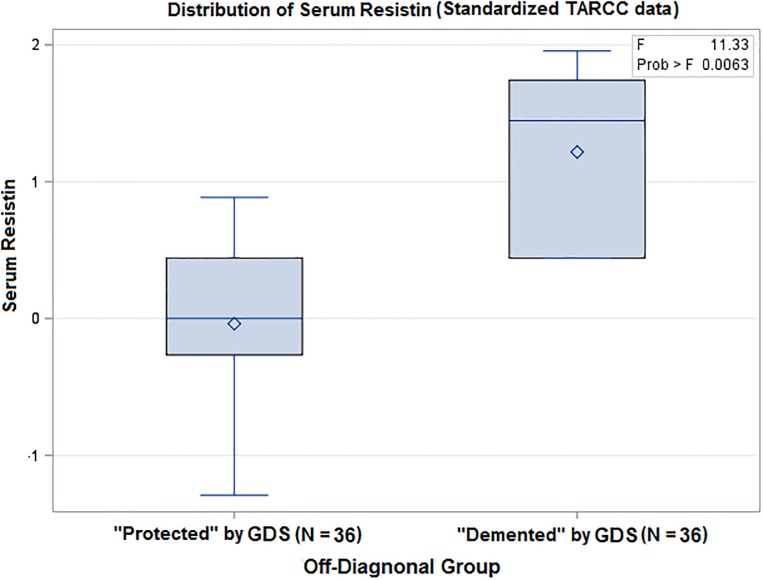
Serum resistin levels are elevated in off-diagonal target cases. *GDS = Geriatric Depression Scale (30 item); TARCC = Texas Alzheimer’s Research and Care Consortium.

The TARCC cases were significantly distributed (by CHI SQ: F = 3324.75 (1), p <0.001). The vast majority were “on-diagonal” given both studies’ selection against clinically depressed cases (Table 2) (Phi Coefficient = 0.957). Regardless, n = 36 TARCC participants, 2.82% of that study’s “AD” cases, were identified as being demented by their depressive symptom burdens. Resistin levels rise significantly across clinical diagnostic groups (Fig 3). The adversely affected off-diagonal group had significantly higher resistin levels in TARCC (by ANOVA, F = 11.33, p = 0.006) (Fig 4). n = 5 (0.8%) of ADNI’s “AD” cases were demented by depression (CHI SQ: F = 1689.1 (1), p <0.001). Plasma resistin levels could not be replicated in ADNI because of the small number of off-diagonal cases.

**Table 2:** “Off-Diagnonal” selection of target cases (TARCC).

	- Unadjusted	+ Unadjusted
- Adjusted	2323	36 Target cases* ≌ 3% of demented participants
+ Adjusted	36	1239

*Target cases revert back to a non-demented d-score when the effect of the GDS score is considered. Such cases are presumed to have traversed the threshold for dementia solely by the effect of their of depressive symtoms.

## Discussion

This analysis supports the hypothesis that clinical “AD” arises from multiple interacting disease processes [47]. Even in the presence of AD-specific biomarkers, dementia yet arise by other means. This may explain the interindividual variability reported in disease-specific biomarkers among cases matched to clinical dementia severity and contribute to so-called “cognitive reserve” [48]. Since the key cognitive factor (i.e., δ) is an aspect of *g*, this would also explain the salience of measures of intelligence to that construct [49].

The present analysis provides proof of concept for an algorithm designed to identify patients converted to dementia by any pre-specified condition or biomarker, and offers a foundation for precision anti-dementia therapy. 1–3% of well-characterized “AD” cases are hereby estimated to be demented solely by the effect of depressive symptoms. This might translate into ≌ 150,000 “AD” cases in the United States (US) [50]. However, our estimates may be lowered by systematic TARCC and ADNI’s selection biases against the recruitment of clinically depressed subjects into their cohorts. The prevalence of such cases in unselected community or primary care dementia cases is likely to be higher than reported here [51].

Our approach can identify cases converted to dementia through depression as individuals. Once appropriate GDS-adjusted and unadjusted d-score thresholds are validated in a well-characterized cohort, they can be applied to an individual’s calculated d-scores by a simple algorithm, e.g., coded for a mobile device app. If the δ homolog being used is indicated by simple bedside measures, or even items of a single test [52], large numbers of patients might be effectively screened. Although infrequently encountered, cases converting to dementia through depression might possibly revert back to non-demented states with treatment of depression’s specific effect. Moreover, this approach identifies only cases who would not be demented without depression’s unique and independent contribution to the δ score. Specific treatment of depressive symptoms broadly in persons with dementia might also improve dementia severity in the far larger fraction of “on-diagonal” dementia cases with depressive symptoms. However, they would not be expected to revert, most likely because they are too impaired by other δ-related dementia risks.

Depression’s unique effect on cognitive performance does not appear to be mediated through neurodegenerative pathologies [53–54]. Instead, the GDS’ association with dementia severity, as measured by δ, can be shown to be mediated through a small set of serum protein biomarkers [19]. Resistin is among those biomarkers, and can be shown to fully attenuate MCI’s 5-year prospective dementia conversion risk in TARCC [55]. Our approach has demonstrably selected for cases with significantly higher serum resistin levels relative to off-diagonal cases “protected” from a dementia diagnosis, i.e., by a GDS score ≤10.

Competition among independent δ-related processes may contribute to “cognitive reserve” among persons at risk for “AD”, and explain depression’s association with reserve. A low GDS score increases the change in δ necessary to effect dementia conversion by any other δ-related process, including AD-specific neurodegeneration. AD-specific neurodegenerative changes have been previously associated with δ [56]. A higher GDS score effectively erodes reserve and might lower the amount of AD-specific pathology required to effect a demented state. Because δ is derived from *g*, this model might also explain reserve’s association with estimators of intelligence.

Our off-diagonal approach might be applied to the effects of age, gender or any other δ-related dementia risk. In their aggregate, these might explain a considerable fraction of so-called “AD” cases and specify which of those comorbidities need to be addressed in individual patients. If all the models were nested over a single cognitive battery (set of indicators), dementia conversions by a number of unique processes could be screened simultaneously by a single cognitive assessment. In all, 20% of well-characterized “AD” cases appear to be demented in the absence of AD-specific biomarkers [57–59]. Their inclusion in clinical trials directed specifically at AD might weaken statistical power to detect effects. The current approach might be applied proactively, i.e., to identify individuals demented uniquely by a biomarker of interest, e.g., amyloidopathy by Positron Emission Tomography (PET), or restrictively, i.e, to exclude cases demented by competing processes.

We have also replicated an earlier report of elevated resistin levels in clinically diagnosed “AD” cases [60]. However, our analysis makes clear that some cases may be pushed into δ’s demented range by resistin’s effect on depressive symptom burden. Although the present algorithm might be used to select individual candidates for anti-dementia therapy by antidepressants, it is currently unknown whether “effective” antidepressant treatment has effects on either δ or resistin. However, cholinesterase-inhibitors have been reported to lower serum resistin [61] and might therefore have a role in the treatment of depressed “AD” cases. Alternatively, their apparent utility in “AD” cases may depend on depressive symptom burden. It is also worth noting that effecting a change in δ will necessitate changes to intelligence. Improvement in domain-specific cognitive performance is orthogonal to *g*/δ *by definition* and is therefore unlikely to effect improvements in dementia severity or functional status [10].

Our study has certain limitations. First, we were limited to the dT2A homolog by our desire to replicate the analysis in ADNI’s data. δ appears to be unlimited by the battery in which it is assessed, but the GDS’ association with those measures might impact its association with the resulting δ homolog composite, and might alter the fraction of off-diagonal cases selected. Second, despite the use of similar biomarker assays by a common vendor, TARCC and ADNI do not overlap precisely either with regard to the biomarkers on their panels or the biomarkers lost to technical issues. We could not attempt replications of all the depression-related mediators identified in reference [19]. Despite their large baseline sample sizes, neither study collected these biomarkers in multiple waves. This restricted us to modeling off-diagonal effects at baseline, temporally close to biomarker measurement, but also to *apriori* selection against clinically recognized cases of depression. Incident depressive symptoms emerging in later waves might have had stronger effects on dT2A (which is available in multiple waves), but in smaller samples (due to attrition) and temporally distant from baseline biomarker assessment. Another limitation is that TARCC’s biomarkers (at least) are associated with batch effects. We can account for that by the introduction of a BIAS construct in SEM [62], but this approach is conducted outside of SEM (which cannot handle ROC analysis). Similarly, we cannot use a BIAS construct to account for differences in the biofluids in which the biomarker is assessed. TARCC is also more affected by ethnicity (ADNI is less diverse). Moreover, some biomarkers are known to have strong ethnicity effects in TARCC (but not resistin) [16, 22].

In summary, we have shown depressive symptoms to uniquely explain dementia conversion in a small fraction of clinically diagnosed “AD” cases from two large well-characterized cohorts. We did so by a novel “off-diagonal” approach using the omnibus transdiagnostic dementia severity measure δ. This analysis provides a means to identify individuals most likely to revert back to non-demented states by the modulation of any pre-specified biomarker or risk factor. The cases selected by this approach, which could be easily automated and /or adapted to brief and convenient cognitive assessments, might then be triaged to specific therapies. The approach can be adapted to any δ-related dementia risk and appears to select for individuals with distinguishable biomarker profiles. We have also replicated an earlier association of clinical “AD” with serum resistin levels and clarified that they may be related through the effect of depressive symptoms on dementia, most likely independently of AD-specific neurodegenerative changes.

## Supporting information

S1 File (Table A) Descriptive statistics by diagnosis (TARCC). (Table B) Descriptive statistics by diagnosis (ADNI).
(DOCX)

## References

[1] Royall DR, Palmer RF, O’Bryant SE. Validation of a latent variable representing the dementing process. Journal of Alzheimer’s Disease, 2012;30:639–649. 10.3233/JAD-2012-120055 22451315

[2] Hughes CP, Berg L, Danziger WL, Coben LA, Martin RL. A new clinical scale for the staging of dementia. British Journal of Psychiatry. 1982;140:566–572. 7104545 10.1192/bjp.140.6.566

[3] Gavett BE, Vudy V, Jeffrey M, John SE, Gurnani A, Adams J. The δ latent dementia phenotype in the NACC UDS: Cross-Validation and Extension. Neuropsychology, 2015;29:344–352. 10.1037/neu0000128 25151112 PMC4340822

[4] Palmer RF, Royall DR. Future dementia status is almost entirely explained by the latent variable δ’s intercept and slope. Journal of Alzheimer’s Disease 2016;49:521–529. 10.3233/JAD-150254 26444763

[5] Koppara A, Wolfsgruber S, Kleineidam L, Schmidtke K, Frölich L, Kurz A, et al The latent dementia phenotype δ is associated with CSF biomarkers of Alzheimer Disease and predicts conversion to AD dementia in subjects with MCI. Journal of Alzheimer’s Disease 2016;49:547–560. 10.3233/JAD-150257 26484902

[6] Peh CX, Abdin E, Vaingankar JA, Verma S, Chua BY, Sagayadevan V, et al Validation of a Latent Construct for Dementia in a Population-Wide Dataset from Singapore. Journal of Alzheimers Disease. 2016;55:823–833.

[7] Andrews SJ, McFall GP, Dixon RA, Cherbuin N, Eramudugolla R, Anstey KJ. Alzheimer’s Environmental and Genetic Risk Scores are differentially associated with general cognitive ability and dementia severity. Alzheimer Disease and Associated Disorders. 2019; in press. 10.1097/WAD.0000000000000292 30681434

[8] John SE, Gurnani AS, Bussell C, Saurman JL, Griffin JW, Gavett BE. The Effectiveness and Unique Contribution of Neuropsychological Tests and the δ Latent Phenotype in the Differential Diagnosis of Dementia in the Uniform Data Set. Neuropsychology. 2016; 30: 946–9609. 10.1037/neu0000315 27797542 PMC5130291

[9] Royall DR, Palmer RF. δ scores predict MCI and AD conversions from non-demented states. Alzheimer’s & Dementia: Diagnosis, Assessment & Disease Monitoring, 2017;6:214–221. 10.1016/j.dadm.2017.02.002 PMC5369695 28378011

[10] Spearman C. General intelligence, objectively determined and measured. American Journal of Psychology. 1904;15:201–293.

[11] Royall DR, Palmer RF. “Executive functions” cannot be distinguished from general intelligence: Two variations on a single theme within a symphony of latent variance. *Frontiers in Behavioral Neuroscience*. 2014;9(369):1–10. 10.3389/fnbeh.2014.00369 PMC4208406 25386125

[12] Royall DR, Matsuoka T, Palmer RF, Kato Y, Taniguchi S, Ogawa M, et al Greater than the sum of its parts: δ Improves upon a battery’s diagnostic performance. Neuropsychology. 2015;29:683–692. 10.1037/neu0000153 25664465

[13] Royall DR, Palmer RF. Construction of a potential telephone assessment of dementia prevalence and severity. Journal of Neuropsychiatry and Clinical Neurosciences. 2018; 30:202–207. 10.1176/appi.neuropsych.17060110 29458281

[14] Royall DR, Palmer RF, Markides KS. Exportation and validation of latent constructs for dementia case- finding in a Mexican-American Population-based Cohort. Journals of Gerontology: Series B: Psychological Science, 2017;72:947–955. 10.1093/geronb/gbw004 PMC5927021 26968639

[15] Royall DR, Palmer RF, Matsuoka T, Kato Y, Taniguchi S, Ogawa M, et al Greater than the sum of its parts: δ can be constructed from item level data. Journal of Alzheimer’s Disease 2016;49:571–579. 10.3233/JAD-150250 26444760

[16] Royall DR, Palmer RF. Thrombopoietin is associated with δ‘s intercept, and only in Non-Hispanic Whites. Alzheimer’s & Dementia: Diagnosis, Assessment & Disease Monitoring. 2016;3:35–42. 10.1016/j.dadm.2016.02.003 PMC4879650 27239547

[17] Royall DR, Al-Rubaye S, Bishnoi R, Palmer RF. Serum protein mediators of dementia and Aging Proper. Aging 2016; 8: 3241–54. 10.18632/aging.101091 27922822 PMC5270666

[18] Royall DR, Al-Rubaye S, Bishnoi R, Palmer RF. Few serum proteins mediate APOE’s association with dementia. PLoS One. 2017;12:e0172268 10.1371/journal.pone.0172268 28291794 PMC5349443

[19] Royall DR, Al-Rubaye S, Bishnoi R, Palmer RF. Serum protein mediators of depression’s association with dementia. PLoS One. 2017;12:e0175790 10.1371/journal.pone.0175790 28594820 PMC5464526

[20] Bishnoi R, Palmer RF, Royall DR. IL-15 as a serum biomarker of Alzheimer disease. PLoS ONE, 2015;10:e0117282. 25710473 10.1371/journal.pone.0117282 PMC4339977

[21] Bishnoi R, Palmer RF, Royall DR. Vitamin D binding protein as a serum biomarker of Alzheimer disease. Journal of Alzheimer’s Disease. 2015;43:37–45. 10.3233/JAD-140042 25079796

[22] Royall DR, Palmer RF. Ethnicity Moderates Dementia’s Biomarkers. Journal of Alzheimer’s Disease. 2015;43:275–287. 10.3233/JAD-140264 25079802

[23] Royall DR, Palmer RF. A δ homolog for dementia case finding with replication in ADNI. Journal of Alzheimer’s Disease. 2019;67:67–79. 10.3233/JAD-171053 30507569

[24] Maixner SM, Burke WJ, Roccaforte WH, Wengel SP, Potter JF. A comparison of two depression scales in a geriatric assessment clinic. *Am J Geriatr Psychiatry* 1995; 3: 60–7. 10.1097/00019442-199524310-00008 28530960

[25] Waring S, O’Bryant SE, Reisch JS, Diaz-Arrastia R, Knebl J, Doody R, for the Texas Alzheimer’s Research Consortium. The Texas Alzheimer’s Research Consortium longitudinal research cohort: Study design and baseline characteristics. *Tex Public Health J* 2008; 60: 9–13.

[26] McKhann D, Drockman D, Folstein M, Katzman R, Price D, Stadlan EM. Clinical diagnosis of Alzheimer’s disease: Report of the NINCDS-ADRDA Work Group. *Neurology*. 1984;34:939–44. 6610841 10.1212/wnl.34.7.939

[27] Weiner MW, Veitch DP. Introduction to special issue: Overview of Alzheimer’s Disease Neuroimaging Initiative. Alz Dementia. 2015;11:730–33. 10.1016/j.jalz.2015.05.007 PMC5536175 26194308

[28] Kaplan EF, Goodglass H, Weintraub S. The Boston Naming Test. Experimental edition. Boston: Kaplan & Goodglass 2nd ed, Lea & Febiger Philadelphia, 1983.

[29] Morris JC, Heyman A, Mohs RC, Hughes JP, vanBelle G, Fullenbaum G, et al The Consortium to Establish a Registry for Alzheimer’s Disease (CERAD). Part I. Clinical and neuropsychological assessment of Alzheimer’s disease. *Neurology*. 1989;39:1159–65. 10.1212/wnl.39.9.1159 2771064

[30] Wechsler D. Wechsler Memory Scale–Third Edition (1997). San Antonio, TX: The Psychological Corporation.

[31] Folstein MF, Folstein SE, McHugh PR. “Mini-mental state”. A practical method for grading the cognitive state of patients for the clinician. *J Psychiatric Res*, 1975;12;189–98. 10.1016/0022-3956(75)90026-6 1202204

[32] Reitan RM. Validity of the Trail Making test as an indicator of organic brain damage. *Percept Motor Skills* 1958;8:271–76.

[33] Lawton MP, Brody EM. Assessment of older people: self-maintaining and instrumental activities of daily living. *Gerontologist* 1969; 9: 179–86. 5349366

[34] Pfeffer RI, Kurosaki TT, Harrah CH, Chance JM, Filos S. Measurement of functional activities in older adults in the community. *J Gerontol*. 1982; 37:323–29. 7069156 10.1093/geronj/37.3.323

[35] Juva K, Mäkelä M, Erkinjuntti T, Sulkava R, Yukoski R, Valvanne J, et al Functional assessment scales in detecting dementia. *Age and Ageing*. 1997;26:393–400. 10.1093/ageing/26.5.393 9351484

[36] Teng E, Becker BW, Woo E, Cummings JL, Lu PH. Subtle deficits in instrumental activities of daily living in subtypes of mild cognitive impairment. *Dementia Geriatr Cog Disorders*. 2010; 30:189–97. 10.1159/000313540 PMC2948658 20798539

[37] O’Bryant SE, Xiao G, Edwards M, Devous M, Gupta VB, Martins R, et al; Texas Alzheimer’s Research and Care Consortium (TARCC). Biomarkers of Alzheimer’s disease among Mexican Americans. *J Alz Dis*. 2013;34:841–49. 10.3233/JAD-122074 PMC3608404 23313927

[38] Sheikh JI, Yesavage JA. Geriatric Depression Scale (GDS): Recent evidence and development of a shorter version. *Clin Gerontologist* 1986; 5: 165–73.

[39] Burke WJ, Houston MJ, Boust SC, Roccaforte WH. Use of the Geriatric Depression Scale in dementia of the Alzheimer type. J Am Geriatr Soc 1989;37:856–60. 2760379 10.1111/j.1532-5415.1989.tb02266.x

[40] Arbuckle, JL. Analysis of Moment Structures-AMOS (Version 7.0) [Computer Program], SPSS, Chicago, 2006.

[41] Schafer JL, Graham JW. Missing data: Our view of the state of the art. *Psychol Methods* 2002; 7: 147–77. 12090408

[42] Graham JW. Missing Data Analysis: Making it work in the real world. *Ann Rev Psychol* 2009; 6: 549–76. 10.1146/annurev.psych.58.110405.085530 18652544

[43] Bollen KA, Long JS. Testing Structural Equation Models. Sage Publications, Thousand Oaks, CA 1993.

[44] Wheaton B, Muthén B, Alwin DF, Summer GF. Assessing reliability and stability in panel models In Heise DR (Ed.) Sociology Methodology San Francisco, CA: Jossey-Bass 1977.

[45] Bentler PM. Comparative fit indexes in structural models. *Psychol Bull* 1990; 107: 238–46. 2320703 10.1037/0033-2909.107.2.238

[46] Browne M, Cudeck R. Alternative ways of assessing model fit In Testing structural equation models, Bollen KA, Long JS, eds. Sage Publications, Thousand Oaks, CA, pp. 136–62, 1993.

[47] White LR, Edland SD, Hemmy LS, Montine KS, Zarow C, Sonnen JA, et al Neuropathologic comorbidity and cognitive impairment in the Nun and Honolulu-Asia Aging Studies. *Neurology* 2016;86:1000–8. 10.1212/WNL.0000000000002480 26888993 PMC4799714

[48] Stern Y, Arenaza-Urquijo EM, Bartrés-Faz D, Belleville S, Cantilon M, Chetelat G, et al.; Reserve, Resilience and Protective Factors PIA Empirical Definitions and Conceptual Frameworks Workgroup. Whitepaper: Defining and investigating cognitive reserve, brain reserve, and brain maintenance. Alzheimers Dement. 2018i: S1552-5260(18)33491-5.

[49] Huang AR, Strombotne KL, Horner EM, Lapham SJ. Adolescent cognitive aptitudes and later-in-life Alzheimer disease and related disorders. JAMA Netw Open. 2018;1(5): e181726 10.1001/jamanetworkopen.2018.1726 30646141 PMC6324501

[50] Alzheimer’s Association. 2018 Alzheimer’s Disease Facts and Figures. Alzheimers Dement 2018;14(3):367–429

[51] Ismail Z, Elbayoumi H, Fischer CE, Hogan DB, Millikin CP, Schweizer T, et al Prevalence of depression in patients with Mild Cognitive Impairment: A systematic review and meta-analysis. *JAMA Psychiatry*. 2017;74:58–67. 10.1001/jamapsychiatry.2016.3162 27893026

[52] Royall DR, Palmer RF, Matsuoka T, Kato Y, Taniguchi S, Ogawa M, et al Greater than the sum of its parts: δ can be constructed from item level data. *J Alz Dis* 2016;49:571–9. 10.3233/JAD-150250 26444760

[53] Royall DR, Palmer RF. Alzheimer pathology does not mediate the association between depressive symptoms and subsequent cognitive decline. *Alz Dis & Dementia* 2013; 9: 318–25. 10.1016/j.jalz.2011.11.009 PMC4459124 23154050

[54] Brendel M, Sauerbeck J, Greven S, Kotz S, Scheiwein F, Blautzik J, et al for the Alzheimer’s Disease Neuroimaging Initiative. Serotonin Selective Reuptake Inhibitor treatment improves cognition and grey matter atrophy but not amyloid burden during two-year follow-up in Mild Cognitive Impairment and Alzheimer’s Disease patients with depressive symptoms. *J Alz Dis*. 2018;65:793–806. 10.3233/JAD-170387 30010116

[55] Royall DR, Al-Rubaye S, Bishnoi R, Palmer RF. Serum protein biomarkers of δ fully mediate multiple AD conversion risks and offer targets for intervention [abstract]. *J Prev Alz Dis*. 2016;3,283.

[56] Gavett BE, John SE, Gurnani AS, Bussell CA, Saurman JL. The role of Alzheimer’s and cerebrovascular pathology in mediating the effects of age, race, and apolipoprotein E genotype on dementia severity in pathologically confirmed Alzheimer’s disease. *J Alz Dis*. 2016;49:531–45. 10.3233/JAD-150252 PMC4858176 26444761

[57] Witte MM, Trzepacz P, Case M, Yu P, Hochstetler H, Quinlivan M, et al Association between clinical measures and florbetapir F18 PET neuroimaging in mild or moderate Alzheimer’s disease dementia. *J Neuropsychiatr Clin Neurosci* 2014;26:214–20. 10.1176/appi.neuropsych.12120402 24618911

[58] Degenhardt EK, Witte MM, Case MG, Yu P, Henley DB, Hochstetler HM, et al Florbetapir F18 PET amyloid neuroimaging and characteristics in patients with mild and moderate Alzheimer Dementia. *Psychosomatics* 2016; 57: 208–16. 10.1016/j.psym.2015.12.002 26892326

[59] Landau SM, Horng A, Fero A, Jagust WJ. Alzheimer’s Disease Neuroimaging Initiative. Amyloid negativity in patients with clinically diagnosed Alzheimer disease and MCI. *Neurology* 2016; 86: 1377–85. 10.1212/WNL.0000000000002576 26968515 PMC4831042

[60] Kizilarslanoğlu MC, Kara Ö, Yeşil Y, Kuyumcu M,E, Öztürk ZA, Cankurtaran M, et al Alzheimer disease, inflammation, and novel inflammatory marker: resistin. *Turk J Med Sci*. 2015;45:1040–6. 26738345

[61] Satapathy SK, Ochani M, Dancho M, Hudson LK, Rosas-Ballina M, Valdes-Ferrer SI, et al Galantamine alleviates inflammation and other obesity-associated complications in high-fat diet-fed mice. *Molecular Med*. 2011;17:599–606. 10.2119/molmed.2011.00083 PMC3146607 21738953

[62] Royall DR, Bishnoi R, Palmer RF. [abstract] Blood-based protein predictors of dementia severity as measured by δ: Replication across biofluids and cohorts. Alzheimer’s & Dementia: The Journal of the Alzheimer’s Association. 2018;14;P649–650.

