# Supplementary material for: Selection for depression-specific dementia cases with replication in two cohorts
Source: PLoS One. 2019 May 31;14(5):e0216413. doi: 10.1371/journal.pone.0216413 (PMC6544211; doi:10.1371/journal.pone.0216413)
Supplement: S1 File — (Table A) Descriptive statistics by diagnosis (TARCC). (Table B) Descriptive statistics by diagnosis (ADNI). (DOCX) [file pone.0216413.s001.docx]

**Supplementary Table A: Descriptive Statistics by Diagnosis (TARCC)**

|  | **TARCC Total**  **Sample**  **N = 3502**  **Mean (SD)** | **TARCC**  **AD**  **N = 1275**  **Mean (SD)** | **TARCC**  **MCI**  **N = 732**  **Mean (SD)** | **TARCC**  **Controls**  **N = 1445**  **Mean (SD)** |
| --- | --- | --- | --- | --- |
| **Gender (%♀)** | 61.6 | 56.2 | 58.0 | 68.3 |
| **Ethnicity (%MA)** | 36.0 | 13.7 | 45.4 | 50.3 |
| **Age** | 70.79 (9.56) | 75.44 (8.41) | 71.34 (8.54) | 66.42 (8.99) |
| **Education** | 13.30 (4.28) | 14.04 (3.71) | 12.73 (4.31) | 12.92 (4.63) |
| **MMSE** | 25.57 (4.73) | 21.49 (4.91) | 26.81 (2.82) | 28.55 (1.91) |
| **Animals** | 14.92 (5.53) | 10.88 (4.66) | 14.85 (4.99) | 17.45 (4.85) |
| **BNT*** | 7.92 (4.27) | 6.59 (3.58) | 7.84 (4.03) | 9.11 (4.58) |
| **CDR-SB** | 2.39 (3.32) | 5.76 (3.29) | 1.24 (0.88) | 0.01 (0.06) |
| **GDS30** | 5.58 (5.23) | 6.06 (5.12) | 6.93 (5.76) | 4.52 (4.80) |
| **LMI** | 7.86 (4.22) | 4.23 (2.53) | 7.67 (3.28) | 10.74 (3.39) |
| **LMII** | 8.22 (4.56) | 3.75 (2.39) | 8.13 (3.42) | 11.69 (3.06) |
| **Trails B (sec)** | 144.24 (84.05) | 210.21 (84.60) | 146.85 (77.30) | 100.62 (46.31) |

*TARCC uses 30 item BNT.

Animals = Animal Naming; BNT = Boston Naming Test; CDR-SB = Clinical Dementia Rating scale “Sum of Boxes”; GDS = 30 item Geriatric Depression Scale; LMI = Wechsler Logical Memory immediate recall; LMII = Wechsler Logical Memory delayed recall; MA = Mexican-American; MMSE = Mini-mental State Exam; SD = standard deviation; TARCC = Texas Alzheimer’s Research and Care Consortium; Trails B = Trail Making Test Part B.

**Supplementary Table B: Descriptive Statistics by Diagnosis (ADNI)**

|  | **ADNI**  **Total**  **Sample**  **N = 1738**  **Mean (SD)** | **ADNI**  **AD**  **N = 342**  **Mean (SD)** | **ADNI**  **MCI***  **N = 978**  **Mean (SD)** | **ADNI**  **Controls**  **N = 417**  **Mean (SD)** |
| --- | --- | --- | --- | --- |
| **Gender (%♀)** | 55.1 | 44.7 | 42.8 | 49.9 |
| **Ethnicity (%MA)** | 3.4 | 3.5 | 3.3 | 3.4 |
| **Age** | 73.8 (7.19) | 75.03 (7.79) | 72.91 (7.42) | 74.76 (5.73) |
| **Education** | 15.91 (2.86) | 15.18 (2.99) | 16.00 (2.82) | 16.28 (2.73) |
| **MMSE** | 27.17(2.67) | 23.22 (2.07) | 27.75 (1.81) | 29.07 (1.12) |
| **Animals** | 17.15 (5.93) | 12.25 (4.98) | 17.39 (5.22) | 20.60 (5.50) |
| **BNT**** | 25.97 (4.51) | 22.24 (6.05) | 26.43 (3.68) | 27.94 (2.66) |
| **CDR-SB** | 1.64 (1.79) | 4.39 (1.67) | 1.36 (0.95 | 0.03 (0.13) |
| **GDS30** | 1.42 (1.40) | 1.65 (1.44) | 1.63 (1.41) | 0.75 (1.12) |
| **LMI** | 9.28 (4.83) | 4.08 (2.80) | 9.10 (3.91) | 13.98 (3.25) |
| **LMII** | 7.07 (5.33) | 1.37 (1.89) | 6.46 (4.10) | 13.18 (3.33) |
| **Trails B (sec)** | 122.23 (75.78) | 191.46 (89.69) | 113.61 (65.42) | 85.68 (43.18) |

* Includes all subtypes and subjective cognitive impairment (SCI)

**ADNI uses 60 item BNT.

ADNI = Alzheimer’s Disease Neuroimaging Initiative; Animals = Animal Naming; BNT = Boston Naming Test; CDR-SB = Clinical Dementia Rating scale “Sum of Boxes”; GDS = 30 item Geriatric Depression Scale; LMI = Wechsler Logical Memory immediate recall; LMII = Wechsler Logical Memory delayed recall; MA = Mexican-American; MMSE = Mini-mental State Exam; SD = standard deviation; Trails B = Trail Making Test Part B.
